# Prevalence of mental disorders and psychological trauma among conflict- affected population in Somalia: a cross-sectional study

**DOI:** 10.3389/fpubh.2023.1219992

**Published:** 2023-09-27

**Authors:** Abdulwahab M Salad, SK Md Mamunur Rahman Malik, James Mwangi Ndithia, Zeynab Noor, Marina Madeo, Mohamed Ibrahim

**Affiliations:** ^1^School of Public Health and Tropical Medicine, Somali National University, Mogadishu, Somalia; ^2^WHO, Somalia Country Office, Mogadishu, Somalia; ^3^Department of Mental Health and Substance Use, Federal Ministry of Health, Mogadishu, Somalia; ^4^School of Social Work, The University of British Columbia, Vancouver, BC, Canada

**Keywords:** Somalia, conflict, trauma, mental disorders, epidemiological patterns, youth, psychiatric status rating scales

## Abstract

**Background:**

Despite the longstanding psychosocial impact of the interactable conflict in Somalia for the last 30 years, there is lack of epidemiological studies of mental health conditions, especially at the population level.

**Objectives:**

The aim of this study is to fill the epidemiological gap and provide population based data on mental health conditions in the South-Central region of Somalia. The specific objectives were: (1) To determine the epidemiological patterns of mental disorders in three sites; Baidoa, Dolow and Kismayo, (2) Understand the socio-demographic characteristics associated with mental health conditions in the study sites, and (3) To assess the correlates between psychological trauma and the mental wellbeing of the population.

**Methods:**

This was a cross-sectional study of 713 respondents recruited from the three sites namely Dolow, Baidoa and Kismayo. Data on sociodemographic characteristics and mental disorders were collected using the MINI and sociodemographic questionnaire. Basic descriptive statistics were used to summarize sociodemographic characteristics. Univariable and multivariable logistic regressions were used to examine factors associated with common mental disorders. Statistical significance was considered at a value of *p* <0.05.

**Results:**

Participants’ mean age was 32.6 (±10.7) years. More than half (58.5%) of the respondents were male. The overall prevalence of common mental disorders was 557 (78.1%) with panic disorder (39.3%), generalized anxiety disorders (34.9%), major depressive episode current (32.1) and PTSD (29.9%). According to the multivariable logistic regression analysis, being male AOR = 1.74 (95%CI = 1.25, 2.42), having a family size of more than 10 members AOR =1.37 (95% CI = 1.00, 1.89), being unemployed AOR = 1.90 (95%CI = 1.18, 3.06), experienced starvation AOR =3.46 (95%CI = 2.23, 5.37), khat use AOR = 5.87 (955 CI, 1.75–19.65), were identified as predicting factors for the common mental disorders among the study participants.

**Conclusion:**

There is a high prevalence of mental disorders with anxiety disorders being the commonest. Findings reflect earlier studies that showed higher rates in conflict and post-conflict settings. It also aligns with past studies in Somalia. As such, there is an urgent need to integrate mental health and psychosocial support within the primary healthcare and other service sectors such as education considering the vast majority of the population are young.

## 1. Introduction

Somalia is a Horn of African nation with approximately 16 million people of which 70% are below the age of 30 years, making it one of the youngest nations in the world (1). Somalia’s population is generally homogenous in terms of language, culture and religious background, with the overwhelming majority speaking the Somali language (2). The country has faced decades of conflict and civil strife, lawlessness and severe disruption of law and order. The country has been without an effective central government since the overthrow of the military rule of Mohamed Siad Barre in 1991 and until the establishment of the current institutional system with the 2012 Provisional Constitution, still extremely fragile.

However, the conflicts and crisis in Somalia has existed for much longer than the past 3 decades of state collapse. It dates back to colonial times when European powers (Britain, France, and Italy) violently partitioned, occupied, and colonized the vast Somali-inhabited Horn of Africa region that includes present-day Somalia, Djibouti and parts of Ethiopia and Kenya (2–4). As such, the longstanding conflict has brought untold suffering, massive loss of lives, gross human rights violations, psychological traumas, and significant displacements that continue to the present day (2, 5).

With the adoption of the 2012 Provisional Constitution, currently, the country is politically and administratively federated with six regional states; Benadir Regional Administration, Puntland, Jubaland, Galmudug, South West and Hirshabelle states. In addition, Somaliland, situated in the north, self-declared independence in 1991 after the collapse of the central government but remains unrecognized internationally. The northern regions of Puntland and Somaliland have over the last two decades enjoyed relative stability as compared to South-Central regions which continues to endure conflict and violence to date (1, 6). The relative stability in the north has contributed to improved security, social, economic and health situations (7).

### Mental health in conflict and post-conflict settings

The global impact of mental, neurological and substance use disorders (MNS) stand at 10% of the general population, but that percentage jumps to more than double at 22% in countries affected by humanitarian crises (8, 9). The majority (75%) of those affected by MNS are in low- and middle-income countries, especially in conflict and post-conflict settings, and lack access to effective mental health services (9, 10). With the high rates of mental health conditions in humanitarian settings, the current climatic crisis compounded by COVID-19 pandemic has only made the mental health situation worse (11). The ongoing climatic shocks has had profound impact on the Somali nation as a large section of the population maintain century old pastoralism lifestyle which is increasingly been threated by recurrent drought. According to UN estimates, over 6 million people been affected by the current drought (12).

The Eastern Mediterranean Region of WHO (EMRO) comprising of 21 countries across Horn of Africa (Somalia and Djibouti), North Africa, Middle East, South Asia (Pakistan and Afghanistan) with a population of nearly 700 million host half of the over 80 million displaced population in the world (11).

In Somalia, the protracted conflict, social unrest and ensued political instability since the fall of the central government in 1991 has left the country in a complex humanitarian situation. The profound impact of political instability, conflict, insecurity, coupled with resultant economic stagnation and poverty, has been a driving force behind the increased rates of mental illness and psychosocial distress across all segments of society, with vulnerable groups such as women and youth most affected (1, 13, 14).

A systematic review of mental health in Somalia (1) estimated that a third or more of the population experienced and or experiencing some form of mental illness. The review also identified that many of the psychosocial problems faced by the people stem from the effects of the protracted conflict, long-term displacements, economic turmoil and social deprivations. While three decades of unstable governance and the collapse of public institutions have worsened access to livelihoods, education, employment and healthcare services. To date, parts of Somalia especially the South-Central region continues to experience significant insecurity and violence. Thus, the continuous conflict and violence, exacerbated by recurring climatic shocks, have deeply fragmented the social fabric of the society and eroded people’s coping mechanisms, community resilience and contributing to the severe psychological distress (1, 6, 15).

Studies have shown significant mental health issues associated with conflict and refugee experiences in diverse settings (1, 15–17). The same is true for Somalis (18–20). Psychological experiences associated with living in conflict and crisis have been classified in the biomedical discourse under the categories of post-traumatic stress disorder (PTSD), anxiety, and depression. Somalis also acknowledge feelings of hopelessness, despair, anxiety, and anguish as part of their symptomatology, despite not explicitly labeling such experiences and symptoms as PTSD or depression (18). The after-effects of trauma among Somalis have also been described in somatic terms, with emphasis on headaches and other unexplained body pains (18, 21).

Despite this, few studies on Somali mental health exist, especially coming out of Somalia. The few studies that have been conducted bear grim results; for instance, a situational analysis by World Health Organization (WHO) in 2010 estimated that one in every three Somalis suffers from some form of mental illness, thus making Somalia one of the countries with the highest rate of mental illness in low-income countries (6). As such, mental illness is a significant health concern in Somalia, a country that has faced decades of political instability, civil unrest, and extreme violence. The prolonged conflict and violence in Somalia have exposed many people to trauma and stress, which are known risk factors for mental illness. Studies showed high exposure to traumatic events, displacement, and significant rates of mental health disorders (1, 6, 13).

Similarly, recent studies (1, 13, 17) showed significant increase in substance use, suicide, anxiety and depression among the Somali population. At the same time highlighting the near total absence of mental health services across the country. The country’s mental health services are close to non-existent with a strikingly low ratio of just 0.5 psychiatric beds per 100,000 population. In contrast, the WHO Eastern Mediterranean Region maintains a ratio of 6.4 beds/100000, and globally, the figure stands at 24 beds/100000 (22). Apart from a handful of inadequately staffed and under-resourced psychiatric hospitals, the country has no community-based mental health services (1, 6, 13). This dire situation leaves the population without adequate support and exacerbates the challenges faced by those suffering from mental health issues.

Equally impacted by the civil war is the loss of essential human resources in health care as most either fled the country or were victims of the war (1, 6). Hence the country is also facing a serious situation of acute and chronic shortage of health professionals. Such a shortage is more profound in the field of mental health where only a handful of professionals provide services across the country (1, 6).

Cultural beliefs and practices can also impact the perception and treatment of mental illness in Somalia. For example, there is widespread belief that mental illness as a result of the evil eye, *jinn*, or other supernatural forces, leading to stigmatization and a reluctance to seek professional help (1, 14). This drives a significant majority to seek traditional and alternative healing services or simply have those with mental illness abandoned or chained at home (1, 6, 14).

Research on mental illness in Somalia is limited, due in part to the ongoing conflict and access constraints, lack of resources, and stigma around mental health. One of the major challenges in studying mental illness in Somalia is the lack of reliable data, due to limited number of public and private health care facilities offering mental health services, and limited surveys, including data that should be routinely collected by the country’s health information system (1). However, a small number of studies conducted provides some insight into the nature of mental illness in Somalia. Most of these studies relied on small sample sizes, or were qualitative in nature, which limited the generalizability of their findings (6, 13, 23, 24).

Although there have been efforts to study and understand the mental health needs for the population in Somalia, there are no population wide studies to provide evidence on the mental health status of the population. This study bridges that gap by undertaking a study of a large size sample using a cross-sectional survey in South-Central Somalia, a first for the country.

The aim of this study was to provide population-based epidemiological data on mental health conditions. The specific objectives were:

1. To determine the epidemiological patterns of mental disorders in Kismayo and Dolow in Jubaland and Baidoa in South West State within South-Central Somalia.
2. To understand the sociodemographic characteristics associated with mental health conditions.
3. To assess the correlates between psychological trauma and the mental wellbeing of the population.

## 2. Methods

### 2.1. Study area

This study was carried out in the three districts mentioned above (Baidoa, Dolow and Kismayo). Baidoa is located approximately 240 kilometers west of Mogadishu. It is characterized by rapid population growth, unplanned urbanization, influx of political refugees, long-standing history of drought and famine.

Kismayo is approximately 500 km southwest of Mogadishu. Following the collapse of central government that accompanied the civil war in 1991, various local militias fought for control of the city. The strategic importance of the port, the land fertility of the Juba region for agricultural cultivation and inter-clan conflict contributed to decades of decades of displacement and distress.

Dolow town, a border town of Somalia and Ethiopia that is located approximately 470 km northwest of Mogadishu. It has often suffered from strained relations between Ethiopia and Somalia, making its residents displaced multiple times. In the last three decades, the town further suffered the predicament of rival militias fighting over territories and invasions by the Ethiopian army (25, 26).

#### Study design, population, and period

This study is a community based cross-sectional study conducted with 713 individuals living in the study sites from October to December 2021. All people aged ≥18 years of both non-displaced and internally displaced people (IDP) were recruited during the study period. Participants were excluded in cases of serious health conditions or disability that impeded their ability to competently consent and or complete the questionnaire.

### 2.2. Study variables

Common mental disorders (CMD) were considered as dependent variables while independent variables included socio-demographic factors such as (age, marital status, education level, family size, and occupation); experience of traumatic events (hunger, combat and witness of killing, lack of shelter and food), and substance use (alcohol, tobacco products, khat and sedatives).

### 2.3. Data collection procedures and quality control

The data was collected through face-to-face interviews using demographic questionnaire and pretested Somali version of Mini International Neuropsychiatric Interview (MINI) version 5.0 to generate sociodemographic, mental disorders, and substance related data. MINI is a short diagnostic tool developed for the assessment of ICD10 (psychiatric disorders). It can be administered in approximately 35–45 min by trained interviewers. The MINI 5.0 comprises of structured psychiatric assessment for all common mental disorders, severe mental health disorders, substance abuse, and suicide risk. This study used the English version of the MINI 5.0 and adapted it to the Somali context. Three authors of this paper, a mental health specialist, a psychologist, and a public health specialist, who are all of Somali origin, collaboratively adapted the English MINI 5.0 to Somali language and cultural context. The MINI questionnaire was then coded in Kobo Collect Software (KoboToolbox, Global) for use on Android operating systems (Google Corporation, Mountain View, CA, USA) by the study team including the research assistants and enumerators. After coding, the research team re-reviewed the Somali translated and adopted MINI 5.0 with focus on understandability, simplicity, and logical sequence of the questions. Eight research assistants (with bachelor in medicine or nursing) were recruited for this study. The research assistants attended a 2-day course on the correct use and administering of the MINI 5.0, in which the research assistants practiced administering and being screened with the MINI 5.0 among themselves.

Further, 60 enumerators (20 for each site) with a minimum bachelor’s degree were recruited for data collection. A two-day workshop was held for all the enumerators by the by the first author and the research assistants to ensure that the enumerators understand the administering of the instruments to the study participants. Enumerators received one-on-one supervision by the research assistants to ensure proper administration of the tool. Next, a pilot study of the MINI 5.0 was conducted, in which the questionnaire was tested for 2 days among the study participants with the supervision of the first author and research team. Enumerators who administered the MINI 5.0 in the piloting were asked for their thoughts on each question, such as, whether the question was obscure or inappropriate or difficult to understand and if so, how they suggested to improve each issue. Following the pilot implementation, the study team improved the MINI 5.0 based on the enumerators’ feedback. Eligible participants were referred to a trained enumerator, who administered the MINI 5.0 and the sociodemographic questionnaire.

### 2.4. Data processing and analysis

The collected data was checked for completeness and consistency and exported into SPSS version 23 for analysis. Summary of descriptive statistics were calculated to explain the socio-demographic characteristics, experience of trauma events, substance related factors and CMD. To determine factors associated with common mental disorders, first bi-variable logistic regression was performed. Subsequently, significant variables in the bi-variable analysis (value of *p* <0.05) were further incorporated into the multi-variable logistic regression. Statistical significance was declared at *p* < 0.05 and the corresponding 95% confidence interval. Chi-square tests were used for categorical data to analyze the differences in levels of mental disorder proportions across socio-demographic characteristics.

## 3. Results

### 3.1. Sociodemographic characteristics of respondents

Table 1 summarizes sociodemographic information collected from the study’s respondents in terms of their residence, age groups, and household characteristics among other variables. The demographic details represent both the non-displaced populations and internally displaced persons (IDPs). Of importance is that most participants were below the age of 34 represented 62.9% of the total study population of 713 while 37% were over 35 years.

**Table 1:** Socio-demographic characteristics (*n* = 713).

Variables	Frequency	Percentage
Sex of the respondent		
Male	417	58.5
Female	296	41.5
Age of the respondent		
15–24	170	23.8
25–34	279	39.1
≥ 35	264	37.0
Marital status		
Married	360	50.5
Single	214	30.0
Divorced	95	13.3
Widowed	44	6.2
Family members		
1–4	153	21.5
5–9	427	59.9
≥ 10	133	18.7
Education level		
Cannot read and write	276	38.7
Quranic school	294	41.2
Secondary grade or less	125	17.5
College or university graduate	18	2.5
Occupational status		
Unemployed	618	86.7
Employed	94	13.2
Study site		
Kismayo	258	36.2
Baidao	225	31.6
Dolow	230	32.3
Area of residence		
Community	576	80.8
IDPs	137	19.2

In respect to levels of education, we looked at various categories as shown in Table 1, and our findings show a low level of formal education, i.e., 39% of the respondents stated that they could not read or write even in their own language while 41% have some form of Islamic education from attending local Quranic schools known as *Dugsi* in the Somali language. *Dugsi* typically involves writing and memorizing Qur’an, the Islamic holy book. Whereas 17.5% attended elementary or high school, and only 2% had acquired college or university education.

We looked further at marital status of the participants and the findings showed 50% were married while 30% reported being single and the remaining classified as either widowed (6%) or divorced (13%). In addition, the majority 78% of the respondents came from a family with five children or more. Overall, significant group differences (*p* < 0.05) were observed in all the variables in relation to the study site; i.e., non-displaced, displaced communities, family size, and age as well as education levels.

### 3.2. Prevalence of mental disorders

We assessed mental health disorders using the various ICD 10 categories. (Table 2) summarizes the prevalence of the most common mental disorders as identified. Overall panic disorder, generalized anxiety disorders, post-traumatic and stress related conditions, and mood disorders appeared to be the most prevalent conditions. Importantly, panic disorders (39%), generalized anxiety disorders (35%), major depressive disorders (32.1%), and PTSD (30%) are the four most common complaints among the study population, while overall, a staggering 78.1% of the participants endorsed experiencing some form of mental or psychological conditions.

**Table 2:** Common mental health disorders among study participants.

Mental health disorders	Category	Frequency	Percentage
Overall common mental health problems	No	156	21.9
	Yes	557	**78.1**
Major Depressive Episode current	No	484	67.9
	Yes	229	**32.1**
Major Depressive Episodes recurrent	No	558	78.3
	Yes	155	**21.7**
Dysthymia current	No	650	91.2
	Yes	63	**8.8**
Suicide Risk	No	555	77.8
	Yes	158	**22.2**
Hypomanic	No	583	81.8
	Yes	130	**18.2**
Manic Episodes	No	572	80.2
	Yes	141	**19.8**
Panic Disorder without Agoraphobia current	No	433	60.7
	Yes	280	**39.3**
Panic Disorder with Agoraphobia current	No	481	67.5
	Yes	232	**32.5**
Post-Traumatic Stress Disorder Current	No	500	70.1
	Yes	213	**29.9**
Mood Disorder with psychotic feature lifetime	No	553	77.6
	Yes	160	**22.4**
Mood Disorder with psychotic features current	No	484	67.9
	Yes	229	**32.1**
Psychotic Disorder Current	No	511	71.7
	Yes	202	**28.3**
Psychotic Disorder Lifetime	No	503	70.5
	Yes	210	**29.5**
Generalized Anxiety Disorder Current	No	464	65.1
	Yes	249	**34.9**

### 3.3. Mental disorders and sociodemographic characteristics among study participants

Results in Table 3A show sociodemographic variables (age, sex, marital status) and (Table 3B) (occupation, residence, site). In our analysis, there was an observed statistically significant results based on age, sex, and marital status (*p* < 0.05) in the presence of manic disorder. Sex and age had a statistically significant (*p* < 0.05) difference with the presence of hypomania, manic episodes, panic disorder with agoraphobia (current), mood disorder with psychotic features (current), psychotic disorder (current), and psychotic disorder (lifetime). Age and marital status showed a significant difference (*p* < 0.05) with depressive episodes (recurrent). In addition, age also appeared statistically significance difference (p < 0.05) with mood disorder with psychotic features (lifetime).

**Table 3A:** Mental disorders stratified by sex, age, and marital status (*n* = 713).

ICD-10 mental disorders	Sex			Age				Marital status				
	Male	Female	value of p	15–24	25–34	≥35	*p*-value	Married	Single	Divorced	Widowed	P-value
Major depressive episode (current)	142 (34.1%)	87 (29.4%)	0.189	49 (28.8%)	90 (32.3%)	90 (34.1%)	0.517	123 (34.2%)	65 (30.4%)	32 (33.7%)	9 (20.5%)	0.279
Major depressive episodes (recurrent)	103 (24.7%)	52 (17.6%)	0.023	38 (22.4%)	60 (21.5%)	57 (21.6%)	0.975	80 (22.2%)	54 (25.2%)	20 (21.1%)	1 (2.3%)	0.010
Dysthymia (current)	41 (9.8%)	22 (7.4%)	0.266	11 (6.5%)	24 (8.6%)	28 (10.6%)	0.328	34 (9.4%)	18 (8.4%)	7 (7.4%)	4 (9.1%)	0.925
Suicide risk	94 (22.5%)	64 (21.6%)	0.771	37 (21.8%)	56 (20.1%)	65 (24.6%)	0.439	75 (20.8%)	51 (23.8%)	19 (20%)	13 (29.5%)	0.501
Hypomanic	91 (21.8%)	39 (13.2%)	0.003	28 (16.5%)	63 (22.6%)	39 (14.8%)	0.049	63 (17.5%)	45 (21%)	20 (21.1%)	2 (4.5%)	0.063
Manic episode	98 (23.5%)	43 (14.5%)	0.003	32 (18.8%)	71 (25.4%)	38 (14.4%)	0.005	65 (18.1%)	55 (25.7%)	19 (20%)	2 (4.5%)	0.008
Panic disorder without agoraphobia (current)	168 (40.3%)	112 (37.8%)	0.509	71 (41.8%)	119 (42.7%)	90 (34.1%)	0.093	128 (35.6%)	105 (49.1%)	36 (37.9%)	11 (25%)	0.002
Panic disorder with agoraphobia (current)	151 (36.2%)	81 (27.4%)	0.013	56 (32.9%)	105 (37.6%)	71 (26.9%)	0.028	107 (29.7%)	88 (41.1%)	30 (31.6%)	7 (15.9%)	0.003
PTSD (current)	128 (30.7%)	85 (28.7%)	0.569	50 (29.4%)	96 (34.4%)	67 (25.4%)	0.071	92 (25.6%)	73 (34.1%)	36 (37.9%)	12 (27.3%)	0.044
Mood disorder with psychotic feature (lifetime)	101 (24.2%)	59 (19.9%)	0.176	38 (22.4%)	76 (27.2%)	46 (17.4%)	0.023	74 (20.6%)	58 (27.1%)	22 (23.2%)	6 (13.6%)	0.145
Mood disorder with psychotic features (current)	150 (36%)	79 (26.7%)	0.009	50 (29.4%)	105 (37.6%)	74 (28%)	0.039	114 (31.7%)	77 (36%)	30 (31.6%)	8 (18.2%)	0.143
Psychotic disorder (current)	135 (32.4%)	67 (22.6%)	0.004	44 (25.9%)	99 (35.5%)	59 (22.3%)	0.002	100 (27.8%)	68 (31.8%)	26 (27.4%)	8 (18.2%)	0.310
Psychotic disorder (lifetime)	140 (33.6%)	70 (23.6%)	0.004	45 (26.5%)	100 (35.8%)	65 (24.6%)	0.010	109 (30.3%)	67 (31.3%)	27 (28.4%)	7 (15.9%)	0.221
Generalized anxiety disorder (current)	145 (34.8%)	104 (35.1%)	0.920	49 (28.8%)	108 (38.7%)	92 (34.8%)	0.103	116 (32.2%)	71 (33.2%)	47 (49.5%)	15 (34.1%)	0.016

**Table 3B:** Mental disorders stratified by employment status, displacement status and site (*n* = 713).

ICD-10 mental disorders	Occupation			Residence area			Site			
	*P*-value	Unemployed	Employed	*P*-value	Community	IDPs	*P*-value	Kismayo	Baidoa	Dolow
Major depressive episode (current)	0.064	206 (33.3%)	23 (24.5%)	0.086	177 (30.7%)	52 (38.0%)	0.103	54 (20.9%)	114 (50.7%)	61 (26.5%)
Dysthymia (current)	0.423	56 (9.1%)	7 (7.4%)	0.608	55 (9.5%)	8 (5.8%)	0.169	22 (8.5%)	17 (7.6%)	24 (10.4%)
Suicide risk	0.839	138 (22.3%)	19 (20.2%)	0.645	123 (21.4%)	35 (25.5%)	0.288	52 (20.2%)	57 (25.3%)	49 (21.3%)
Hypomanic	0.090	108 (17.5%)	22 (23.4%)	0.166	109 (18.9%)	21 (15.3%)	0.327	45 (17.4%)	69 (30.7%)	16 (7%)
Manic episode	0.063	126 (20.4%)	15 (16%)	0.315	117 (20.3%)	24 (17.5%)	0.460	47 (18.2%)	78 (34.7%)	16 (7%)
Panic disorder	0.001	246 (39.8%)	34 (36.2%)	0.501	242 (42%)	38 (27.7%)	0.002	107 (41.5%)	119 (52.9%)	54 (23.5%)
Post-traumatic stress disorder (current)	0.010	190 (30.7%)	23 (24.5%)	0.216	187 (32.5%)	26 (19%)	0.002	71 (27.5%)	99 (44%)	43 (18.7%)
Psychotic disorder (current)	0.039	181 (29.3%)	21 (22.3%)	0.164	174 (30.2%)	28 (20.4%)	0.023	62 (24.0%)	100 (44.4%)	40 (17.4%)
Psychotic disorder (lifetime)	0.015	185 (29.9%)	25 (26.6%)	0.508	176 (30.6%)	34 (24.8%)	0.185	64 (24.8%)	110 (48.9%)	36 (15.7%)
Generalized anxiety disorder (current)	0.291	224 (36.2%)	25 (26.6%)	0.068	199 (34.5%)	50 (36.5%)	0.667	79 (30.6%)	108 (48%)	62 (27%)

Marital status is a moderating variable (*p* < 0.05) in panic disorder without agoraphobia (current), PTSD (current), and generalized anxiety disorder (current). There was no observable significant difference by age, sex, and marital status in major depressive episode (current), dysthymia (current), and suicide risk.

In Table 3B, largely, significant differences by site and occupation (*p* < 0.05) were observed in three disorders, namely, panic, post-traumatic stress, and psychotic disorder. Psychotic disorder (lifetime) was statistically significant different with occupation at *p* < 0.05. There were significant site differences (*p* < 0.05) in most disorders among participants who were from Dolow and Kismayo with lower proportion of mental disorders compared to those from Baidoa.

### 3.4. Factors associated with common mental disorder.

In this study, the overall prevalence of common mental disorders (CMD) was found to be 78.1%. It was higher in males 335 (47%) than females 222 (31%). In bivariable analysis, being male, aged between 15–24 or 25–34 years, unemployed, having family size above or equal 10 members and experienced starvation were associated with CMD. According to the multivariable logistic regression analysis, variable with value of p of less than 0.5 were considered as statistically significant associated with CMD. Accordingly, sex, marital status, occupation, family size, experience of ill-health without access to medical care, experiences of combat situation, bodily harm, starvation, use of sedatives or sleeping pills, khat, and tobacco products had significant associations with CMD. The odds of experiencing CMD for unemployed participants were 1.90 times higher (AOR = 1.90, 95% CI = 1.18–3.06) than employed. Those who had family size of equal or more than 10 members were 3.3 times more likely to develop CMD (AOR = 3.3, 95% CI = 1.90–5.72) compared with those who had family size of 1–4 members. The odds of having CMD among study participants who experienced starvation was 3.46 times (AOR = 3.46, 95% CI = 2.23–5.37) higher as compared to their counterparts. Participants who use khat were 5.87 times more likely to have CMD (AOR = 5.87, 955 CI, 1.75–19.65) than participants who did not use. Participants who were physically harmed or beaten were 95% more likely to have CMD (AOR = 1.95, 95% CI = 1.23–3.09) compared to those who did not experience the same (Table 4).

**Table 4:** Factors associated with any mental health disorders among the study participants.

Variable	MHDs		COR	AOR	*p*-value
	No	Yes			
Sex					
Male	82	335	4.08 (3.20, 5.201)	1.74 (1.25, 2.42)*	**0.001**
Female	74	222	**1**	**1**	
Age					
15–24	39	131	3.35 (2.34, 4.80)	1.12 (0.71, 1.78)	0.605
25–34	57	222	3.89 (2.91, 5.21)	1.40 (0.96, 2.05)	0.077
≥35	60	204	**1**	**1**	
Marital status					
Married	95	265	**1**	**1**	
Single	29	185	6.37 (4.31, 9.43)	2.38 (1.45, 3.89)*	**0.001**
Divorced	22	73	3.31 (2.06, 5.34)	1.63 (0.96, 2.76)	0.069
widowed	10	34	3.40 (1.68, 6.88)	2.04 (0.97, 4.28)	0.059
Family size					
1–4	33	120	**1**	**1**	
5–9	106	321	3.02 (2.43, 3.77)	1.37 (1.00, 1.89) **	**0.049**
≥10	17	116	6.82 (4.10, 11.35)	3.30 (1.90, 5.72)*	**0.000**
Education level					
Cannot read and write	59	217	3.67 (2.75, 4.90)	1.09 (0.60, 1.95)	0.768
Quranic Madarasa	65	229	3.52 (2.67, 4.64)	1.01 (0.59, 1.71)	0.969
secondary grade or less	31	94	3.03 (2.02, 4.55)	0.95 (0.53, 1.72)	0.886
College or university graduate	1	17	**1**	**1**	
Occupation					
Unemployed	126	492	3.90 (3.21, 4.74)	1.90 (1.18, 3.06)*	**0.008**
Employed	30	64	**1**	**1**	
Residence area					
Community	118	458	**1**	**1**	
IDPs	38	99	2.60 (1.79, 3.78)	0.69 (0.44, 1.10)	0.122
Lack of shelter					
No	73	235	**1**	**1**	
Yes	83	322	3.88 (3.04, 4.93)	0.77 (0.51, 1.17)	0.233
Ill health without access to medical care					
No	50	79	**1**	**1**	
Yes	106	478	4.50 (3.65, 5.56)	3.14 (2.00, 4.93)*	**0.000**
Combat situation					
No	133	331	**1**	**1**	
Yes	23	226	9.82 (6.39, 15.09)	2.82 (1.72, 4.62)*	**0.000**
Beatings (bodily harm)					
No	127	334	**1**	**1**	
Yes	29	223	7.69 (5.22, 11.32)	1.95 (1.23, 3.09)*	**0.004**
Witness killing/murder					
No	151	497	**1**	**1**	
Yes	5	60	12.00 (4.81, 29.88)	1.69 (0.61, 4.65)	0.305
Experience Starvation					
No	127	285	**1**	**1**	
Yes	29	272	9.37 (6.39, 13.75)	3.46 (2.23, 5.37)*	**0.000**
Tobacco products					
No	110	326	**1**	**1**	
Yes	46	231	5.02 (3.66, 6.89)	1.82 (1.26, 2.62)*	**0.001**
Alcoholic beverages					
No	147	477	**1**	**1**	
Yes	9	80	8.88 (4.46, 17.70)	0.77 (0.27, 2.183)	0.626
Sedatives or Sleeping Pills					
No	125	323	**1**	**1**	
Yes	31	234	7.54 (5.19, 10.97)	4.02 (2.66, 6.08)*	**0.000**
Khat chewing					
No	153	512	**1**	**1**	
Yes	3	45	15.00 (4.66, 48.26)	5.87 (1.75, 19.65)*	**0.004**

### 3.5. Traumatic experience and substance use

In this table, we looked at the co-morbidity between various mental health conditions ie anxiety, depression and PTSD and substance use. More than one third of the study participants (42.2%) experienced starvation. Similarly, one third of the participants (35.3%) reported that they had been exposed to physical bodily harm or beatings. Of the total of 713 participants, 277 (38.8%) reported using tobacco products, and 265 (37.2%) reported using sedatives or sleeping pills. In addition, most of the study participants (81.9%) reported that they have experienced ill health without access to medical care (see Table 5).

**Table 5:** Traumatic experience and substance use.

No	Substances use	Categories	Frequencies	Percentages
**1**	Alcoholic beverages (beer, wine, spirits, etc.)	No	624	87.5
		Yes	89	**12.5**
**2**	Tobacco products (cigarettes, chewing tobacco, cigars, etc.)	No	436	61.2
		Yes	277	**38.8**
**4**	Sedatives or Sleeping Pills (Valium, Serepax, Rohypnol, etc.)	No	448	62.8
**5**	Khat chewing	Yes	265	**37.2**
**6**		No	665	93.3
7	Ill health without access to medical care	No	129	18.1
8		Yes	584	81.9
9	Beating to the body	No	461	64.7
10		Yes	252	35.3
11	Experience Starvation	No	412	57.8
12		Yes	301	42.2
13	Witness killing/murder	No	648	90.9
14		Yes	65	9.1
15	Witness killing/murder	No	648	90.9
		Yes	65	9.1

## 4. Discussion

To our understanding, this is the first large scale cross-sectional study on mental illness in Somalia that covers a large geographical portion of the country. The study attempted to partly bridge an important epidemiological hole in Somalia in understanding the link between psychological trauma and mental health in the context of a complex and intractable conflict. Importantly, it also adds to the knowledge base in similar conflict and post-conflict settings.

Some of the demographic highlights are that the population is young with the vast majority (62.9%) below the age of 34 years. These statistics reflect earlier population estimates that show Somalia having one of the youngest populations of about 70% (23). Regarding mental health conditions, the findings show a significantly high level of anxiety (34.9%), PTSD (30%), and depression (32.1%) among the study population. This again is likely the reflection of the many years of conflict, displacement, and associated psychological distress which shows significant presence of trauma-related conditions among the Somali population and supported by other studies done in Somalia (1, 6, 13, 24). A recent study (17) showed high level of PTSD (32%) among IDP population in Mogadishu, Somalia which is almost similar 30% to the findings of this study. While studies in Colombia (27), Uganda (28), Kashmir (29), Ethiopia (30) and Syria (31) showed elevated levels of CMDs especially depression, anxiety and PTSD in conflict and post-conflict settings. However, the rates vary across these settings and the difference in rates of mental health conditions across conflict-related settings could be due to a variety reasons such as; use of different study tools, sampling techniques, cultural differences in illness narratives, nature of humanitarian situation as well personal and communal experiences of trauma (inter-communal, interpersonal and or intimate partner violence). Nevertheless, this and other studies in conflict and humanitarian settings show the impact of trauma and associated psychosocial stressors on the psychosocial wellbeing of the affected individuals, families and communities.

For the findings of this study, if all types of anxiety disorders are combined, then they outstrip all disorders, suggesting the state of fear and uncertainty of the Somali population.

## 5. Conclusion and recommendations

In conclusion, the study confirms the high prevalence of mental health disorders, particularly anxiety, PTSD, and depression among the study population. The armed conflict is likely to end but the psychological impact will likely be an intergenerational challenge if not comprehensively and adequately addressed. The long-term effects of adverse childhood experiences have been well documented and show children who experience significant childhood challenges such as violence, hunger and death of parents may develop long-term physical and psychological health conditions such as hypertension, diabetes, mental illness, and substance abuse (32–34).

Thus, this study shows the urgent need to address the mental health needs of the population, especially the youth who represent the majority both in the sample and in the general population. For a population so young, traumatized, uneducated, and hugely unemployed, Somalia’s crisis is far from over and requires comprehensive approach to addressing trauma and resultant psychological distress in a culturally appropriate framework that involves inter- and multi-sectoral stakeholders that cuts across health, social, educational, political, and economic services.

Expanding the provision of mental health care and psychosocial support is a dire need in Somalia, and given the acute and chronic shortage of mental health specialists (psychiatric nurses, psychologists, social workers and psychiatrists), it is unlikely in the foreseeable future that Somalia will be able to train sufficient mental health specialists (8). Therefore, it is prudent, realistic and strategic to adopt a task-sharing model in which non-specialists provide mental health services integrated into primary health care (35). With the huge demand for mental health services, the country and its development partners need to strategically address the treatment gap through a task-sharing model in order to address the unmet needs, strengthen and utilize existing health infrastructure and workforce, while increasing community outreach and support through enlisting more community health workers (36).

## 6. Strengths and limitations

The strengths of this study were the use of standard psychometric tools that were translated as per WHO guidelines and the community engagements from services users, policy makers and community members. The online data collection using GPS enhanced technology reduced inconsistency and able to address any technicalities. The MINI assessment tool that we utilized has been cross-culturally used in other settings in the past but has not been validated in the Somalia context as such we translated in the local Somali language, tested and piloted in the context of South-Central Somalia.

Thus, a significant limitation of this study is, that the assessment tool has not yet been validated in the population under consideration. This is a task for future studies. The project was also significantly impacted by the global COVID-19 pandemic, as it was implemented at the height of the pandemic when movements were restricted thus delaying the implementation as well the data collection.

## Data availability statement

The data supporting the findings of this study is available at Somali National University. Requests can be made to the lead author AS.

## Ethics statement

The studies involving humans were approved by The Somali National University Ethics Review Board. The studies were conducted in accordance with the local legislation and institutional requirements. The participants provided their written informed consent to participate in this study.

## Author contributions

The SM author was the overall lead of the project and contributed to the overall success of the project, while AS, JN, ZN, MM, and MI undertook the trainings, supervisions, data collection, analysis, and write-up of the manuscripts. The MI author also coordinated the manuscript development and is the corresponding author. All authors contributed to the article and approved the submitted version.
