# Supplementary material for: Prevalence of mental disorders and psychological trauma among conflict- affected population in Somalia: a cross-sectional study
Source: Front Public Health. 2023 Sep 27;11:1219992. doi: 10.3389/fpubh.2023.1219992 (PMC10565346; doi:10.3389/fpubh.2023.1219992)
Supplement: Supplementary file 1 [file Data_Sheet_1.PDF]

Abbreviations

ACE	Adverse Childhood Experiences
PTSD	Post Traumatic Stress Disorder
WHO	World Health Organization
SNU	Somali National University
IDP	Internally Displaced Persons
MINI	Mini International Neuropsychiatric Interview
ICD	International Classification of Diseases
IRB	Institutional Review Board
mhGAP	Mental Health Gap Action Program
